# The Genetic, Morphological, and Physiological Characterization of a Dark Larval Cuticle Mutation in the Butterfly, *Bicyclus anynana*

**DOI:** 10.1371/journal.pone.0011563

**Published:** 2010-07-14

**Authors:** Ashley Bear, Ariel Simons, Erica Westerman, Antónia Monteiro

**Affiliations:** Department of Ecology and Evolutionary Biology, Yale University, New Haven, Connecticut, United States of America; Michigan State University, United States of America

## Abstract

Studies on insect melanism have greatly contributed to our understanding of natural selection and the ultimate factors influencing the evolution of darkly pigmented phenotypes. Research on several species of melanic lepidopteran larvae have found that low levels of circulating juvenile hormone (JH) titers are associated with a melanic phenotype, suggesting that genetic changes in the JH biosynthetic pathway give rise to increased deposition of melanin granules in the cuticle in this group. But does melanism arise through different molecular mechanisms in different species? The present study reports on a *Bicyclus anynana* (Lepidoptera: Nymphalidae) *dark larvae* single locus mutation, in which larvae exhibit a darker cuticle relative to wild type. Unlike other lepidopteran melanic larvae mutations, this one is autosomal recessive and does not appear to involve a deficiency in JH titers. Unlike JH deficiency mutants, dark larvae mutants display similar growth rates and sexual behaviors as wild type, and topical application of a JH analogue failed to rescue the wild type cuticular coloration. Finally, transmission electron microscopy showed that sclerotization or deposition of diffuse melanin, rather than deposition of melanin granules, produces the dark coloration found in the cuticle of this species. We conclude that different molecular mechanisms underlie larval melanism in different species of Lepidoptera.

## Introduction

Melanism, the occurrence of variants that are mostly or completely dark in pigmentation, is widespread in nature and has been featured in classic studies that have greatly contributed to our understanding of natural selection in the wild [1], [2]. Researchers interested in the evolution of melanism have asked whether the melanic phenotypes exhibited by different taxa are produced via the same or different molecular mechanism. Studies on the molecular basis of melanism in vertebrates have found that different genetic mechanisms can produce melanic phenotypes in different taxa [3]. While the molecular basis of melanism in invertebrates has been more limited in taxonomic range, extensive research on *Drosophila* and in some Lepidopterans has shown that the same is true of invertebrate taxa [4]–[10].

While research in *Drosophila* has exclusively focused on mutations in genes from the melanin biochemical pathway [5]–[9], melanism in Lepidoptera appears to be produced through changes in hormone physiology as well as changes in pigment enzymes. Initially, studies on the molecular basis of melanism in Lepidoptera took a physiological approach and investigated the hormonal basis of melanism in the *Manduca sexta* “black” (*bl*) larval mutant. A series of studies on this mutant identified an association between low levels of the insect juvenile hormone (JH) and the melanic phenotype [11], [12]. These results are consistent with work on the larvae of several other lepidopteran species, in which a melanic phenotype is produced as a plastic response to the environment. In these studies, as well as those conducted on the *bl* mutant, topical treatment of melanic individuals with JH or a JH analog rescued the lighter phenotype in subsequent larval instars [4], [11], [13]–[14]. Furthermore, studies in *M. sexta* revealed that reduction of JH (through the removal of the corpora allata) led to a two-fold increase in dopa decarboxylase (DDC), the enzyme that converts dopa into dopamine, which in turn led to increased deposition of pre-melanin granules in the cuticle [15]. When the cuticle of three other species of melanic lepidopteran larvae (*Celerio euphorbiae, Papilio machaon,* and *Phalera bucephala*) were imaged using light and transmission electron microscopy (TEM), researchers found that melanin granules in the cuticle produced the melanic phenotypes in these larvae [16]. Taken together, these studies suggest that mutations influencing JH production are likely to contribute to larval melanism in Lepidoptera via disruption of this hormone's repressive effect on the deposition of melanin granules in the cuticle. More recently, however, studies have used knowledge of the genetic basis of melanism in *D. melanogaster* to identify the genetic basis of melanism in the *Bombyx mori* “sooty” larval mutant. This approach has shown that mutations in *ebony*, a gene in the melanin biochemical pathway, produces the melanic phenotype. From the above we can conclude that melanism in the Lepidoptera can arise through different genetic mechanisms.

The current study investigated the genetic, physiological, and morphological characteristics of a newly discovered lepidopteran melanic larval mutant and found no evidence that JH, nor melanin granules, are involved in producing the melanic phenotype. We report on a “*dark-larvae*” mutation (*dl*) in *Bicyclus anynana* (Lepidoptera: Nymphalidae), which arose spontaneously in 2006 in a few individuals within the colony stock of *B. anynana* maintained in the lab. The mutants exhibit a darker larval cuticle color relative to wild-type (wt) larvae, but are indistinguishable from wt as pupae and adults. After breeding from dl individuals for several generations, a stock was produced that was essentially breeding true. A series of genetic crosses were performed to characterize whether the *dl* mutation is 1) recessive, co-dominant or dominant; 2) autosomal or sex-linked; and 3) controlled by one or more loci. In order to investigate whether the *dl* mutation disrupts the JH synthesis pathway, Ultra performance Liquid Chromatography coupled to Mass Spectrometry (UPLC-MS) was used to compare JH titers between dl and wt. In addition, fourth instar dl and wt larva were topically treated with pyriproxyfen (a JH analog) in order to test whether artificially increasing JH titers would affect the degree of cuticular melanization in the fifth instar. JH deficiencies in other Lepidopteran species have been shown to reduce adult size, increase development time, reduce fecundity, and alter reproductive and courtship behaviors [2], [12], [17]–[18]. If the *dl* mutation reduces JH titers, the dl mutants would likely exhibit some, if not all, of these traits. Thus, adult size and development time were compared between dl and wt individuals, and mating experiments were performed in which mating latency and fecundity in dl and wt were recorded. In order to characterize the mutation at the morphological level, light microscopy and TEM were used to image the cuticle at a range of magnifications. Finally, we explored a possible adaptive value for the dl mutants. Some melanic insect species are known to exhibit a higher resistance to desiccation than their wild type counterparts [2]. This has been proposed to be an adaptive quality favoring the evolution of melanism. In order to address whether the dl phenotype confers such a potential adaptive quality, desiccation resistance was measured in the dl and compared with that of wt larvae.

The results of these experiments indicate that *dl* is a single locus, autosomal recessive mutation; that JH deficiency is not responsible for producing the melanic mutant phenotype in *B. anynana*; that dl individuals have comparable development times, size at maturity, and mating behavior relative to wt individuals; and that the melanic phenotype of *B. anynana* is not produced by the presence of melanin granules, but rather, by other pigmentation processes such as sclerotization or deposition of diffuse melanin in the cuticle. These results are different from those found in other studies of melanic Lepidoptera, adding to the idea that similar dark larval phenotypes can be produced in different species by different molecular mechanisms.

## Materials and Methods

### Butterfly husbandry

wt and dl stocks were reared inside a walk-in climate chamber at 28°C, 80% humidity, and 12 h: 12 h light: dark photoperiod. Larvae were fed on young corn plants and adults were fed on mashed banana.

### Quantifying the dl phenotype

In order to document changes in larval coloration through ontogeny, ten second instar larvae (first instars were deemed too fragile to handle) were removed from the plants in each cage and reared in isolation inside small plastic 4-ounce Solo cups, supplied with cuttings of corn leaves. Larvae were photographed every two days until they became pupae.

Photographs were taken with a Nikon AS-15 camera using a 1/8 second shutter speed, 100 ISO and 11 F. A Canon impact macro lens was attached and positioned 12 cm from a uniform beige plastic mat onto which larvae were placed. Before each photograph the larva was given time to uncurl and stretch out allowing the entire dorsal cuticle to be seen. A gray card for color correction, ruler, and label (caterpillar number and family) were included in each picture.

**Figure 1:**
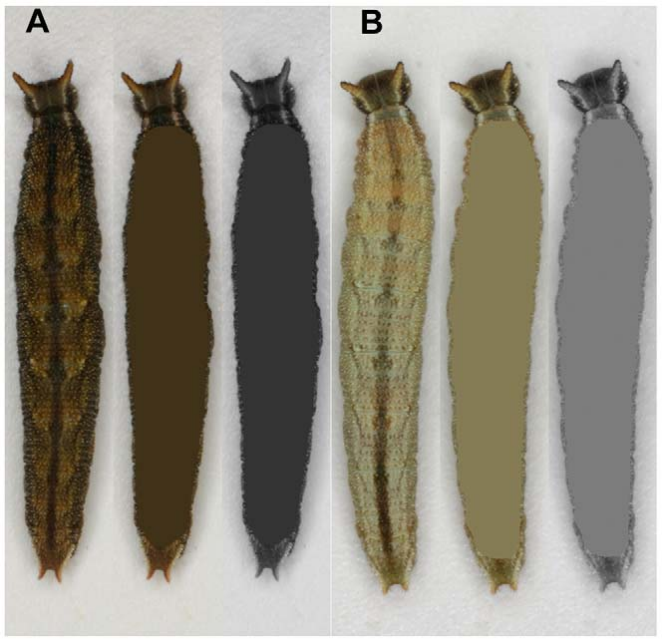
Method of measuring the cuticle brightness value (B-value) of caterpillars. **a.** 5th instar dl. **b**. 5^th^ instar wt. Both original images have been separated from the background using the “lasso” tool, averaged using the “average” tool, and then converted to gray scale in Adobe Photoshop.

These pictures were compiled into panels, where four photos were assembled of each individual, one per larval instar (2^nd^. 3^rd^, 4^th^, and 5^th^). From each family, five representative individuals were scored for their brightness values (B-values) using tools in Adobe Photoshop CS3. Using the “lasso” tool in Photoshop, the outline of each larva was separately selected from the background and averaged using the “blur/average” filter (Fig. 1). Once every larva's color had been individually averaged the entire image was converted to grayscale. Using the “color picker” tool, the B-value was recorded for each larva using a scale of 0 (black) to 100 (white).

To test for differences in larval brightness within families and between cross types, a general lineal model (GLM) was used in which cross type was used as a fixed factor and family as a random factor. Separate analyses were performed for each larval instar. A scatter plot of the brightness values for each larval instar was generated to identify when differences in larval coloration start to appear during larval development. SPSS (Mac version 16) was used for the analysis and graphs.

### Determining the *dl* inheritance pattern

To determine whether *dl* is a recessive, co-dominant, or dominant allele, a series of single pair crosses were performed between dl and wt individuals, and were compared to crosses between dl x dl and wt x wt individuals using the cuticle color evaluation tools described above. Ten replicate cages of each type of cross were set up (dl x dl, wt x dl, and wt x wt). Hybrid crosses consisted of a dl female and a wt male. To address whether *dl* is sex-linked a set of reciprocal hybrid crosses were also performed. If the *dl* mutation is on the Z chromosome, then all female offspring from this cross (dl male crossed with wt female) should be dark, whereas female offspring from the other hybrid cross should be wt. *dl* is not on the W chromosome because dl females are WZ and show the same coloration as dl males (ZZ, and thus not carrying a W chromosome). To assess whether the *dl* mutation is at a single or multiple loci, thirteen single pair backcrosses were performed between dl virgin females and F1 hybrid males (offspring of dl females and wt males).

For all of the crosses described above, each pair was given a fresh corn plant as an oviposition substrate. When larvae were observed on the corn plant, the plant was removed and placed in a mesh larval rearing sleeve. Plants from each backcross were checked every day for the presence of fifth instar larva (the dl phenotype is most apparent at the fifth instar) and both dl and wt fifth instar larva were counted. A chi-squared analysis was run for each cross in order to determine whether the ratio of dl to wt offspring from each backcross differed significantly from a 1∶1 ratio, which is expected only when the dl phenotype is produced by a mutation at a single locus.

### Topical application of JH analogue (JHA)

A series of topical JH application experiments were conducted to determine whether the dl phenotype is produced by changes in JH titers. Studies on *M*. *sexta* and *Papilio xuthus* have shown that there is a limited developmental window in which topical applications of JHA will effect pigmentation in subsequent instars, and that this window varies depending on the species [11], [19]. Topical JHA was applied to dl in varying doses and at different developmental time points in order to observe whether artificially increasing JHA titers would lead to the appearance of a wt phenotype in successive larval instars. wt individuals were also topically treated with JHA.

Each day at 10 a.m. and 4∶00 p.m. larval cages were inspected for newly ecdysed 4th instar larva. These were collected and placed in separate rearing cups. Topical pyriproxyfen (Sigma, 34174-100M), a juvenile hormone analog (JHA), was applied to these larvae at different concentrations and at different time points in development. Larvae in each treatment group had 1 µl of pyriproxyfen solution (or acetone alone) applied to the first thoracic segment. The length of time until the larvae molted into the 5th instar was recorded for each experiment and 5th instar larva were photographed in order to measure any change in cuticle phenotype as a result of the JHA treatments. Length of the 4^th^ instar was also measured because JH is a key hormone in determining larval molting; larvae molt into each successive instar when JH has dropped below a critical threshold [20]. Therefore, treatment with topical JH was expected to delay ecdysis into the 5^th^ instar. Larvae were monitored through to pupation.

#### Experiment I

The aim of the first topical JHA experiment was to identify a physiological correct dose of JHA while keeping the time of JHA application constant. A range of doses was used because this is the first study in which topical JHA has been applied to *B*. *anynana* larvae. The range of chosen doses paralleled those used on close relatives of *B*. *anynana*, or used in adult *B. anynana* in other studies [12], [19], [21]–[24]. For these experiments we used dl larvae between 0 and 20 hours into the 4^th^ instar. This is the time window of JH sensitivity in *P. xuthus,* the closest relative of *B*. *anynana* from which JH sensitivity data is known [19]. Larvae were separated into six different experimental groups. Four treatment groups received different doses of JHA (treatment a: 0.01 µg/µl, treatment b: 1.0 µg/µl, treatment c: 5.0 µg/µl, treatment d: 10.0 µg/µl), one control group received only acetone without JHA, and the other control group was untreated. Because the two highest doses (5.0 µg/µl and 10.0 µg/µl) proved fatal to all larvae in these treatment groups, lower doses were used in all subsequent experiments.

#### Experiment II

In *P. xuthus*, addition of JHA makes 5^th^ instar larvae retain the dark larval color pattern phenotype characteristic of the 4th instar [19]. Thus, in order to test whether artificially increasing JH titers in wt individuals would produce a dl phenotype in the 5^th^ instar, wt individuals 0–20 hours into the 4^th^ instar, were separated into two treatment groups; one received 1.0 µg/µl JHA dissolved in acetone, and one received acetone alone.

#### Experiments III, IV, V

In order to try to identify the developmental window of JH sensitivity in *B. anynana*, topical JHA was applied in a dose of 1.0 µg/µl to dl larvae at three different time intervals: 24–44 hours (Experiment III), 48–68 hours (Experiment IV), and 72–92 (Experiment V) after ecdysis into the 4^th^ instar.

#### Experiment VI

This experiment tested whether a later developmental period, when dl are undergoing head cap apolysis before molting into the 5^th^ instar, was a sensitive period to JH application. dl larvae were separated into two treatment groups; one received a dose of 0.001 µg/µl JHA in acetone, and one received acetone alone. This dose and this time point in development were used because they were shown to be effective in producing a wt phenotype in *M. sexta bl* mutants [11], [12,]. This time point in development has also been shown to be important for melanization in *S. litura* [25].

In each experiment, the statistical program R was used for analysis and graphs.

### Pleiotropic effects of the *dl* mutation

In order to quantify differences in development time between dl and wt, eggs were collected during a four-hour period from each of the adult colony cages and reared to adulthood. Pupation day and pupal weight on that day were recorded for each group (dl and wt).

Differences in mating latency and female fecundity were measured in single pair crosses between virgin homozygous dl males and females (*dl/dl*), heterozygous males and females (*dl/+*) exhibiting the wt phenotype, and wt males and females (*+/+*). Because homozygous dl and heterozygous wt individuals were siblings (both were offspring of a *dl/+* and *dl/dl* back-cross) the possible negative effects of inbreeding on mating success and/or fecundity was shared by these two groups. In order to estimate mating latency, the genitalia of 4–5 day old virgin males was dusted with fluorescent powder [25] and then each male was placed in a cylindrical hanging cage containing a 4–5 day old virgin female. A Petri dish containing mashed banana was also provided. Each day thereafter the female's genitalia was checked for the presence of fluorescent powder, an indication that mating took place. In order to estimate fecundity, the pair was provided with a corn plant as an oviposition substrate as soon as fluorescent powder was found on the female genitalia. Plants were then checked daily for eggs and/or larva.

In an attempt to infer the adaptive potential of the dl phenotype, desiccation resistance was compared between dl and wt. Weight was used as a proxy for desiccation as in Parkash and Ramniwas [26]. dl and wt fifth instar individuals were weighed and then placed individually in a sealed falcon tube containing 4 g of silica gel (a desiccant). After 36 hours the larvae were removed from the tubes and weighed again. In order to control for weight loss due to food deprivation, wt and dl controls were weighed and placed in sealed falcon tubes without silica gel for 36 hours, after which time they were weighed as well. R was used for analysis and graphs.

### Light microscopy

Pieces of cuticle were dissected from the dorsal abdominal segment (between the first and second prolegs) of dl and wt fifth instar larvae, in PBS solution on ice, and then mounted on slides. Cuticles were photographed with a Q Imaging Retiga Ex*i* camera attached to a Nikon Eclipse 90i fluorescence microscope.

### Transmission electron microscopy

Pieces of cuticle of dl and wt fifth instar larvae, dissected as above, were sequentially dehydrated in 10-minute washes with 70% and100% ethanol. Samples were then washed 3 times (5 minute washes) with propylene oxide, and subsequently incubated in a series of different solutions. First for 1 hour in 1∶1 Embed-812: propylene oxide, then for 12 hours in 2∶1 Embed-812:propylene oxide, then for 2 hours in pure Embed-812, and finally embedded in pure Embed-812 for 12 hours. Transverse sections of wild type and mutant cuticle were produced using a MT-1 ultra-microtome and a Diatome diamond knife. Section thickness was determined by section interference color, and all sections used had interference colors of gold or silver (600 to 750Å). Sections were then stained for 10 minutes in uranyl acetate, washed in deionized water, stained for 5 minutes in lead citrate, and washed in deionized water. Samples were viewed under a Zeiss EM-900, and imaged with a MegaView III Soft Imaging System.

### Measuring JH titers

Hemolymph was extracted from 4^th^ instar wt and dl larvae undergoing head cap apolysis, following the protocol of Westerlund and Hoffmann [27]. This is a JH sensitive period in *M. sexta,* when melanin is deposited in the new cuticle of the molting larva. Samples were then submitted along with the commercially available version of JH III (Biochemika 59992) to the Keck Facility Mass Spectrometry Resource Facility at Yale for UPLC-MS quantitation. Using the transitions determined experimentally from the standard, the samples were loaded into a Waters nanoAcquity UPLC. The samples were desalted on a Symmetry C18 180 micron X 20 mm trap column, and then separated on an BEH C18 75 micron X 150 mm analytical column, and introduced into an Applied Biosystems 4000 QTRAP by nanoflow ESMS. Trapping was done at 5 µl/min, 98% Buffer A (100% water, 0.1% formic acid) for 3 min. Small molecule separation was performed at 400 nl/min with Buffer A: 100% water, 0.1% formic acid and Buffer B: 100% CH_3_CN, 0.1% formic acid. A linear gradient (30 minutes) was run with 2% buffer B at initial conditions, 40% B at 30 minutes, and 85% B at 30.33 minutes. Transitions were monitored for a maximum of 100 milliseconds, at unit resolution. Quantitation was done using Multiquant 1.1 software from Applied Biosystems, using an external calibration. The external calibration was generated from a standard curve of the JH III molecule run immediately prior to the samples.

## Results

### Quantification of the *dl* phenotype

**Figure 2:**
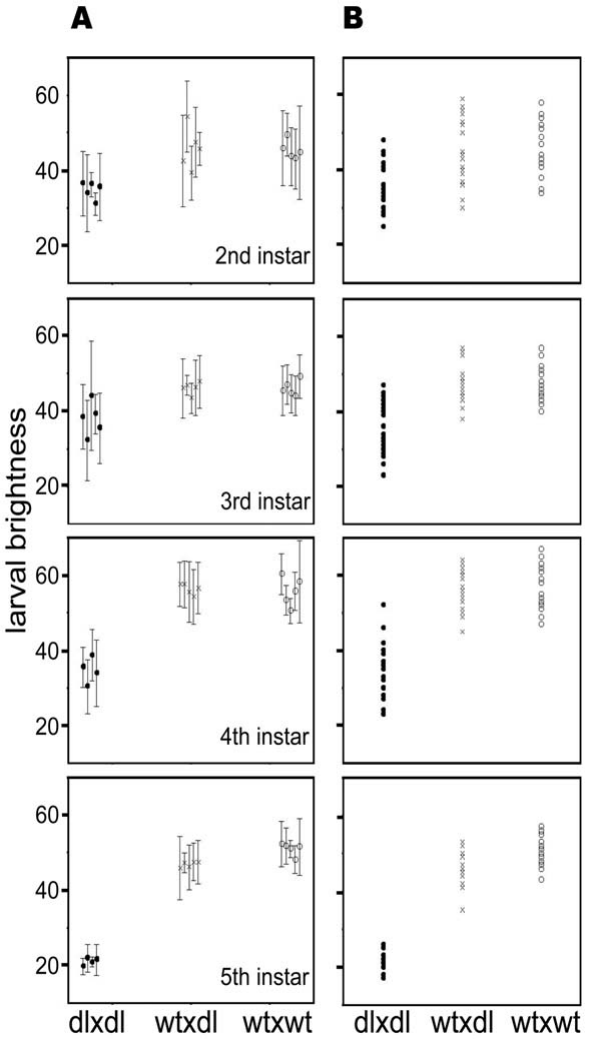
Graphical representation of B-values of offspring from three types of parental cross. **a.** Average B-values (± SD) of offspring from three types of parental cross. Each cross is represented by five families arranged vertically by instar. **b.** Scatter plot to show overlap and spread of B-values for individuals from the families of the three types of parental cross.

**Figure 3:**
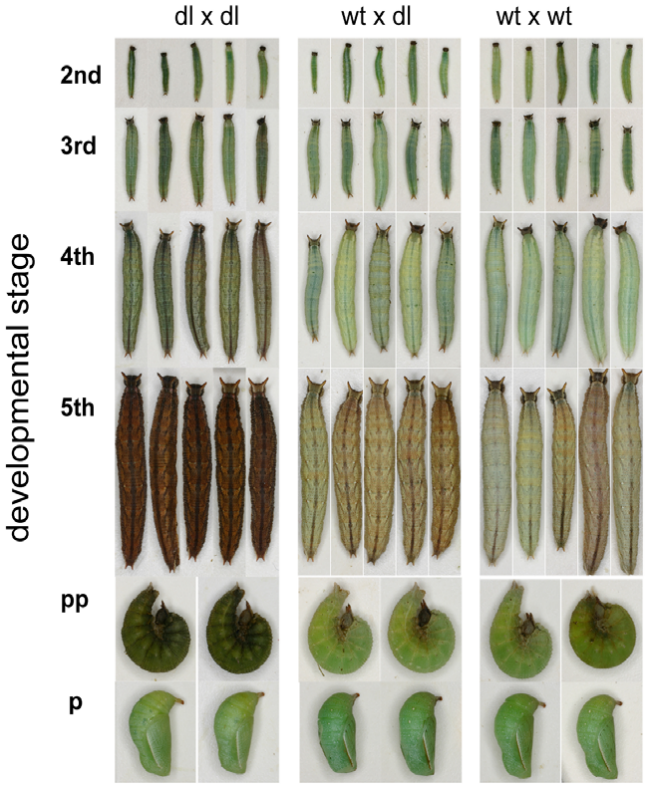
Representative photographs of larval families from the three types of cross. Columns represent siblings from the same family and rows represent the same individual photographed as a 2^nd^, 3^rd^, 4^th^ and 5^th^ instar, as a prepupa (pp) and as a pupa (p).

dl mutants are significantly darker than wt and heterozygous larvae at each of the larval stages examined (Fig. 2) (Table 1). Variance in brightness values was highest in the 4th instar, and dl larvae are at their darkest during the fifth instar (Fig. 2). When dl reached the pupal and adult stages, however, they were visually indistinguishable from wt (Fig. 3).

**Table 1:** Analyses of variance comparing cuticle brightness (average B-values) between dl homozygotes, heterozygotes, and wt homozygotes across 4 larval instars.

instar	B value dl x dl	N	B value wt x dl	N	B value wt x wt	N	F	P
2nd	34.76	25	45.96	25	45.54	24	19.84	0
3rd	38.04	25	46.24	25	46.24	25	14.6	0
4th	35.68	22	56.36	25	55.72	25	87.75	0
5th	21.1	20	46.96	25	51.04	25	390.54	0

N  =  total number of individuals measured.

### Inheritance pattern of *dl*

The crosses performed revealed that the *dl* mutation is a recessive mutation. The cuticle coloration of offspring of wt crossed with dl is indistinguishable from that of offspring from wt crosses (Fig 3). There is also no evidence for a sex-linked mutation given that female offspring from both types of reciprocal crosses (dl male x wt female and wt male x dl female) had a wt phenotype. In twelve backcross families the ratio of dl to wt offspring did not differ significantly from a 1∶1 ratio, suggesting that the *dl* mutation lies at a single locus (Table 2). In two families, however, the ratio of dl to wt differed significantly from a 1∶1 ratio, and it is unclear why this was the case. Since the ratio in the majority of the crosses did not differ significantly from 1∶1, it supports the hypothesis that the *dl* mutation lies at a single locus.

**Table 2:** Chi-square tests applied to the frequencies of dl and wt offspring from twelve backcrosses between *dl*/*dl* and *dl/wt* parents, with an expected 1∶1 ratio.

Family Number	#WT	#DL	Chi squared	p
1	94	61	7.03	0.008
2	6	7	0.077	0.782
3	8	6	0.28	0.593
4	90	49	12.1	0.001
5	39	33	0.5	0.48
6	88	69	2.3	0.129
7	21	24	0.2	0.655
8	88	91	0.05	0.823
9	41	33	0.865	0.325
10	43	31	1.95	0.163
11	28	43	3.17	0.075
12	86	81	0.15	0.699
13	12	14	0.154	0.695
14	38	34	0.222	0.637

### Topical application of JHA

**Figure 4:**
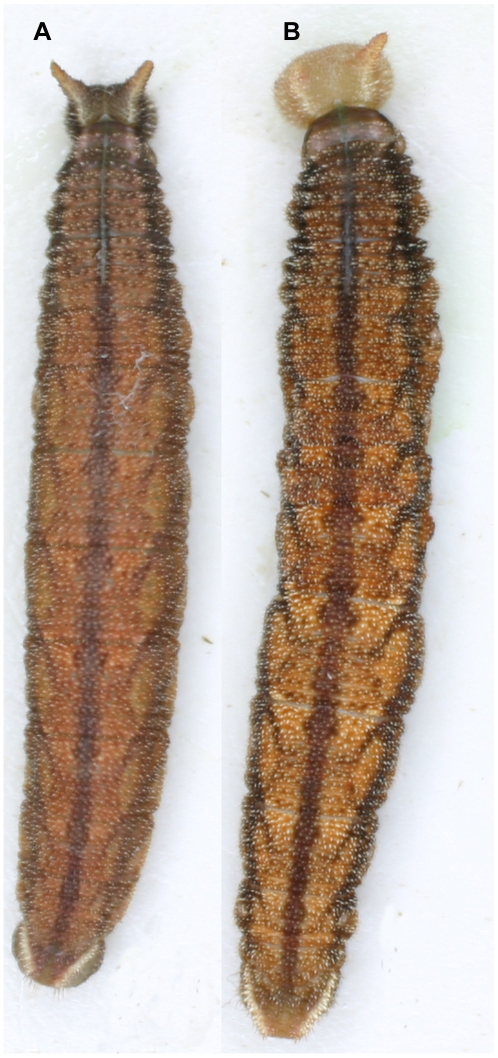
Topical application of JHA to dl leads to a supernumerary instar. **a**. normal 5^th^ instar larva that was treated with acetone. **b**. 6^th^ supernumerary instar larva that was treated with JHA on the 4^th^ day of the 4^th^ instar.

There was no significant difference between the untreated control group and the control group receiving topical treatment with acetone alone (F_1,18_ = 3.69, p = 0.071). Therefore, the acetone control data was used in all analyses. Topical treatment with JHA had no effect on larval coloration in the 5th instar (Table 3). Application of JHA also did not increase the length of the 4^th^ instar (Table 3), but in all experiments, except those in which JHA was applied within 20 hours into the 4^th^ instar, 5^th^ instar larvae molted into a 6^th^ supernumerary instar (Fig. 4). In all cases in which larvae molted into a supernumerary instar, the larvae either died while in the process of head cap apolysis into the 6^th^ instar or a few days later.

**Table 3:** Results of topical Juvenile Hormone analogue (JHA) application on larval cuticle brightness (B-value) in the fifth instar and on the length of the fourth instar.

						Avg B-value (±95% CI)					Avg Length of instar (±95% CI) in days			
Experiment	Strain	Dose of JHA (µg/µl)	N	DTI (hrs)	N	JHA	N	A ctrl	F	p	JHA	A ctrl	F	p
I: treatment a	dl	0.01	10	0–20	10	26.6±5.2	10	25.9±1.9	0.06	0.81	5.3±0.76	4.6±0.32	0.67	0.42
I: treatment b	dl	1	10	0–20	10	28.6±2.2	10	25.9±1.9	3.39	0.08	5.8±0.76	4.6±0.32	2.94	0.1
I: treatment c	dl	5	10	0–20	10	24.1±2.6	10	25.9±1.9	1.2	0.29	5.5±0.73	4.6±0.32	1.22	0.28
I: treatment d	dl	10	10	0–20	10	25.3±2.9	10	25.9±1.9	0.12	0.74	5.4±0.60	4.6±0.32	1	0.33
II	wt	1	5	0–20	5	57.9±4.9	5	54.4±4.1	0.16	0.7	4.8±0.39	4.7±0.30	1.08	0.31
III	dl	1	5	24–44	5	26.4±2.4	5	25.8±3.6	3.2	0.11	4.8±0.40	5.6±0.80	0.06	0.82
IV	dl	1	5	48–68	5	27.8±6.0	5	24.6±3.3	0.33	0.58	4.4±0.50	4.6±0.50	0.84	0.39
V	dl	1	5	72–92	5	25.6±2.5	5	26.8±7.7	4.5	0.07	4.2±0.40	4.8±0.40	0.08	0.79
VI	dl	1	10	HCA	10	24.5±6.3	10	28.3±3.4	0	1	5.0±0.00	5.0±0.00	3.09	0.1

F statistics represent results from Analysis of Variance to test for significant differences across experimental and control treatments in larval color and development time. Experiment; experiment number. DTI; developmental interval when JHA was applied. A ctrl; Acetone control. N; numbers treated. JHA; experimental groups treated with JHA. HCA; time of head cap apolysis.

### Pleiotropic effects of the *dl* mutation

There was no significant difference in the length of time it took dl and wt to develop from an egg to a pupa (26.74 days ±1.56 for wt, 25.93 days ±2.18 for dl; F_1,70_ = 0.08, p = 0.774**)** and there was no significant difference in pupal weight between wt and dl (0.15 g ±0.033 for wt and 0.16 g ±0.024 for dl; F_1,70_ = 1.88, p = 0.175). *dl/dl*, *dl*/*+*, and *+/+* virgin pairs all mated within 28 hours of being placed together. Only 75% of *dl/dl* and *dl/w+* matings resulted in fertile offspring, while 100 percent of *+/+* matings resulted in fertile offspring. Inbreeding has been found to negatively affect fecundity in this species, so it is likely that the reduced fecundity in the *dl*/*d l*and *dl/+* crosses was due to inbreeding due to the small founder population of mutants [28]. Alternatively, reduced fecundity may be a negative pleiotropic effect of the *dl* mutant allele.

A two-way ANOVA revealed that larval color (whether dl or wt) had no significant effect on desiccation resistance (F_1,16_ = 1.01, p = 0.329), while group (experimental or control) had a significant effect on degree of desiccation (F_1,16_ = 19.81, p<0.001).

### Light Microscopy

**Figure 5:**
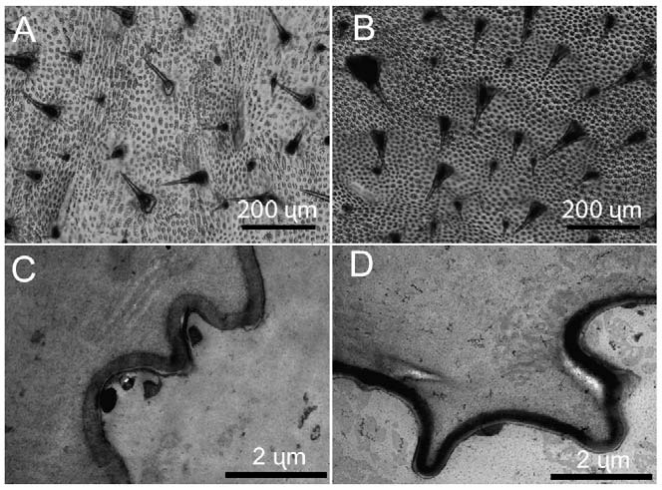
Light microscope and TEM imaging of dl and wt cuticles. **a,b.** Light micrograph of the dorsal cuticle of a fifth instar wt (a) and dl (b) larvae showing darkly pigmented bristles and star-like structures with concentrated pigmentation. **c,d**. TEM micrograph of dorsal cuticle of fifth instar wt (c) and dl (d) showing a star-like structure containing a homogeneous layer of diffuse pigment that is darker in dl (d).

The cuticle of dl is not homogeneously darker, but pigmentation appears to be concentrated in small, stellate structures in the epicuticle (Fig. 5a,b).

### Transmission electron microscopy

Imaging with TEM showed that there are no melanin granules in the *B. anynana* larval cuticle. Rather, the epicuticle appears to contain a distal homogeneous layer of dark pigment (Fig. 5d). The stellate epicuticular structures and the homogeneous appearance of the pigment are found in both wt and dl but the degree of pigmentation is greater in dl.

### Measuring JH titers

UPLC-MS quantitation of JH revealed no significant differences in JH titers between 4^th^ instar wt and dl larvae undergoing head cap apolysis (F_1,2_ = 0.48**,** p = 0.562).

## Discussion

The current study described the phenotype and inheritance pattern of the dl mutation, tested the hypothesis that the dl phenotype is produced by a JH deficiency, looked for pleiotropic effects associated with the mutation, examined a possible adaptive role for the mutation, and reported on the nature of the pigmentation in the dl larval cuticle. The dl phenotype was expressed in all larval instars examined and in the prepupal stage, but lost during pupation and adulthood. Genetic crosses revealed that the *dl* mutation is autosomal recessive and lies at a single locus. Topical applications of JHA failed to produce a lighter phenotype, and no difference was found in JH III titers between dl and wt individuals molting into the fifth instar. There were also no pleiotropic effects found in development time and reproductive behavior or physiology, and the dl phenotype does not appear to confer an adaptive advantage in desiccation resistance. Finally, imaging with a light microscope showed that the dl mutants have darkly pigmented cuticular stellate structures, and imaging with TEM showed that pigmentation in these areas is diffuse and does not resemble melanin granules.

The results of this study indicate that the inheritance pattern, the role of JH, the lack of pleiotropic effects, and the physical nature of the cuticular pigmentation in the dl mutant are different from some previously studied melanic lepidopteran larvae. In particular, the single locus, autosomal recessive inheritance pattern of the *dl* mutation differs from the *M*. *sexta bl* mutation, which is sex linked [12]. Also, while melanic phenotypes in *M. sexta*, *Anticarsia gemmatalis,* and *Cephonodes hylas* larvae can be changed to lighter phenotypes by topical application of JHA, this was found not to be the case for dl [4], [11], [13]–[14]. Predictably, and unlike *M. sexta,* there was no detectable difference in JH III titers in dl mutants undergoing head cap apolysis [12]. Also, unlike other melanic insects, the dl mutant did not exhibit any apparent pleiotropic effects in size, development time, reproduction, or dessication resistance [2], [12], [17]. When imaged using light microscopy and TEM, *C. euphorbiae, P. machaon,* and *P. bucephala* melanic larvae were found to have deposition of melanin granules in the cuticle [15]. Melanin granules have also been described in *M. sexta bl* larvae [16]. In contrast, dl appear to have increased levels of diffuse pigmentation, concentrated in stellate discrete structures, that does not resemble melanin granules.

While these results suggest that the dl phenotype is not due to changes in JH signaling, epidermal-specific changes in the expression of members of the JH receptor complex could potentially be involved. While this explanation remains a possibility, it seems unlikely given that all but null mutations should still respond to artificially increased JH titers compensating for lower expression levels of the receptor. Furthermore, the only effect topical JHA applications had on dl was to stimulate molting into a supernumerary instar, which suggests that JHA was able to effectively stimulate receptors in the epidermis.

Currently, it is not clear what kind of pigmentation process is responsible for the dl phenotype. Due to the homogeneity of the pigmentation at TEM resolution, sclerotization or deposition of diffuse melanin in the cuticle are the most likely causes [15]. Sclerotization and melanization are offshoots of the same initial molecular pathway, and the enzyme N-acetyltransferase, plays a central role in switching between the two outcomes by converting dopamine to the sclerotizing agent N-acetyldopamine [29]. One possible future approach to distinguish sclerotization from melanization in dl would be to compare the expression of the gene coding for N-acetyltransferase between dl and wt. Another approach would be to inject dl with inhibitors of Dopa-decarboxylase, an enzyme required for melanization, but not sclerotization [30], and observe whether this prevents cuticle darkening in dl. If this were the case, then there would be strong reason to believe that deposition of diffuse melanin in the cuticle produces the dl phenotype.

Recent studies in *Bombyx mori* and *D. melanogaster* suggest that alterations to the genes *ebony* and *yellow* underlie some melanic phenotypes in insects. Linkage analysis in *B. mori* showed that an autosomal recessive mutation, sooty (so), results in the production of nonsense *ebony* proteins and in a darker larval color [10]. In *D. melanogaster*, polymorphism in the *ebony* gene is associated with natural variation in pigmentation in the cuticle of adult flies [8]. In laboratory experiments, lower levels of *ebony* expression produce darker thoracic trident pigmentation [9], whereas increased expression of *yellow* is associated with a more melanic phenotype [7]. Since the ebony protein is an enzyme that converts dopamine into N-β-alanyl-dopamine (NBAD), a precursor for a light pigment in many insects, it is possible that in ebony mutants, dopamine is not converted into NBAD, and rather, is recruited into the melanin pathway. The exact role that yellow plays in the melanin pathway is currently unclear [10]. These studies, and those in *M. sexta*, imply that dark phenotypes in insects can be produced by at least three independent mechanisms: up regulation of the gene yellow, down regulation of the gene ebony, or changes in the expression of genes in the JH pathway. In the current study we found no evidence that the dl phenotype is produced by changes in the JH pathway. Therefore, it is possible that a mutation that decreases expression of the *B. anynana* orthologue of *ebony* or increases expression of the *yellow* orthologue could produce the dl phenotype. A future identification of the *dl* allele via linkage mapping would not only improve our understanding of genetic basis of melanism in insects, but might also hold promise in *B*. *anynana* transgenic studies [31]. The apparent lack of negative pleiotropic effects associated with this allele make it potentially very useful as a visible transgenic marker. By inserting the wt copy of this allele into dl mutant embryos, it would be possible to readily identify transgenic individuals at the larval stages of development.

In summary, the genetic, physiological and morphological data we gathered in this study support the idea that larval melanism in insects arises through different molecular mechanisms in different species.
